# Odor Perception and Descriptions of Rose-Scented Geranium *Pelargonium graveolens* ‘Dr. Westerlund’–Sensory and Chemical Analyses

**DOI:** 10.3390/molecules28114511

**Published:** 2023-06-02

**Authors:** Karin Wendin, Anna Maria Pálsdóttir, Sara Spendrup, Lennart Mårtensson

**Affiliations:** 1 Faculty of Natural Sciences, Kristianstad University, SE-291 88 Kristianstad, Sweden; lennart.martensson@hkr.se; 2 Department of Food Science, University of Copenhagen, Rolighedsvej 26, DK-1958 Frederiksberg C, Denmark; 3 Department of People and Society, Swedish University of Agricultural Sciences, P.O. Box 190, SE-234 22 Lomma, Sweden

**Keywords:** sensory profile, chemical profile, gas chromatography, odor identification, Dr. Westerlund, *Pelargonium graveolens*

## Abstract

A recent study found that the natural scent from the rose-scented geranium Pelargonium graveolens ‘Dr. Westerlund’ had positive effects on stress reduction. Essential oils from many pelargonium species are known to have phytochemical properties and pharmacological activities. No study has, so far, explored and identified the chemical compounds and the sensory perception of these compounds in ‘Dr. Westerlund’ plants. Such knowledge would be an important contribution to an increased understanding of the effects of plants’ chemical odor properties on human well-being, and link this to the expressed perceived scents. This study aimed to identify the sensory profile and suggest responsible chemical compounds of *Pelargonium graveolens* ‘Dr. Westerlund’. The sensory and chemical analysis results revealed sensory profiles of Pelargonium graveolens ‘Dr. Westerlund’s and provided suggestions for the chemical compounds attributed to the sensory profiles. Further studies are recommended to investigate the correlation between volatile compounds and possible stress reduction in humans.

## 1. Introduction

The positive impact of nature on human health and well-being is presently gaining momentum and has been studied from numerous perspectives. The phenomenon is thus presently receiving great scientific and societal interest, and several researchers have explored the health benefits of people who reside in natural environments [1,2], nature’s capacity to reduce stress [3,4], as well as to restore cognitive functions and facilitate mental recovery, thus, improving psychological health [5,6,7]. As of today, several positive health effects have been identified and linked to different components of contact with nature: (a) reducing exposure to noise and air pollution; (b) facilitating mental and physical stress reduction; (c) increasing physical activity; (d) strengthening the immune system; and (e) enhancing social contacts [8]. Further, several studies on “forest bathing” have also reported health benefits through sensory stimulation, an integrated process including all the senses, i.e., sight, smell, touch, sound, and taste [9,10]. When separating the visual, auditory, and olfactory stimuli on stress reduction, odors, as opposed to sight and sound, were viewed as having a more profound effect on stress [4]. This finding is congruent with previous results supporting stress-reducing effects, e.g., “green odours” [11] and the odor of lavender [12]. Findings suggest that the auditive has been identified as a dominating sense. Still, it is of importance to pay further attention to additional sensory nature-originated stimuli supporting human health and well-being, such as in stress reduction.

Studies have additionally indicated that olfaction and odors have a significant impact on human memory [13,14,15,16]. In a recent study by Pálsdóttir et al. [17], it was found that nature scent (soil, wood, dried straw, conifer needles, and vegetation), specifically in rose-scented geranium *Pelargonium graveolens* ‘Dr. Westerlund’, elicited positive associations, emotions, and physical reactions among participants recovering from stress-related mental disorders. In this specific study, the pelargonium plants were made available as potted plants with which participants could engage in activities, such as propagation, re-potting, watering, and trimming the plants. The findings further suggest that experiencing the smell of pelargonium (described as, e.g., apple, peppermint, orange, rose, eucalyptus, and citrus) may facilitate perceived stress reduction and support mental recovery. Yet, to our understanding, no study has explored and identified the chemical compounds and the sensory perception of these compounds in ‘Dr. Westerlund’. Such knowledge would be an important contribution to further increase an understanding of the effects that plants’ chemical odor properties can have on human health and well-being, and link this to the expressed perceived scents. The plant species *Pelargonium* sp. comprises a wide variety of aromatic plants and are known to have phytochemical properties and pharmacological activities. Particularly, the rose-scented species *Pelargonium graveolens* is a commercially important plant in the form of essential oils and extracts in the perfumery and cosmetic industries [18].

A variety of chemical compounds are produced from plants during growth and development. Some of the compounds are volatile and named biogenic volatile organic compounds (BVOCs). They play important roles in plant growth, development, reproduction, and defence, as well as being highly responsible for the odor composition of the plants [19]. Flowers, stems, and leaves release BVOCs to attract insects and animals for their own reproduction; however, as described previously, these compounds have also been shown to promote health and well-being [9]. 

Sensory science is a methodology “to evoke, measure, analyze and interpret those responses to products as perceived through the senses of sight, smell, touch, taste, and hearing” [20]. By using the analytical sensory methodology, i.e., non-subjective methods, especially the method of quantitative descriptive analysis, the odor profile of *Pelargonium graveolens* ‘Dr. Westerlund’ could be developed. This is conducted by developing the descriptors of the odor, i.e., of the BVOCs, and then by the use of a scale, evaluating the intensity of each descriptor. The panelists are selected due to their highly sensitive senses and then trained on the task. Due to the low concentration (ppb) in the air, the amounts of BVOCs collected directly from the air are insufficient for chemical analysis. Sampling techniques, including solid-phase micro-extraction (SPME) and static headspace (SHS), coupled with gas chromatography and mass spectrometry (GCMS), help to collect and analyze the BVOCs emitted from the leaves. These techniques are considered simple and fast. Solid-phase microextraction (SPME) has been widely employed in odor and flavor analysis because of the fast and solvent-free sample preparation, its high sensitivity, and the possibility of automation. This technique is routinely used in headspace (HS) analysis of volatiles from different sample types: environmental, biological, and foodstuffs [21]. Relating the descriptors from the sensory odor profile to the identified chemical compounds will increase the understanding of which BVOCs in *Pelargonium graveolens* ‘Dr. Westerlund’ may have an impact on human well-being and health. The odor profile may additionally function as an input to the horticultural industry by increasing customers’ understanding of the breadth of plant odor and how these are linked to health and well-being.

This study aimed to develop a sensory profile by the use of quantitative descriptive analysis to describe the sensory perception and find suggestions for the responsible chemical compounds by a qualitative analysis of the volatile compounds, analyzed by gas chromatography and mass spectrometry, present in *Pelargonium graveolens* ‘Dr. Westerlund’ as a potted plant, leaves, and stem cuttings. This study included 16 plants analyzed in duplicate.

## 2. Results

### 2.1. Sensory Analysis

**Figure 1:**
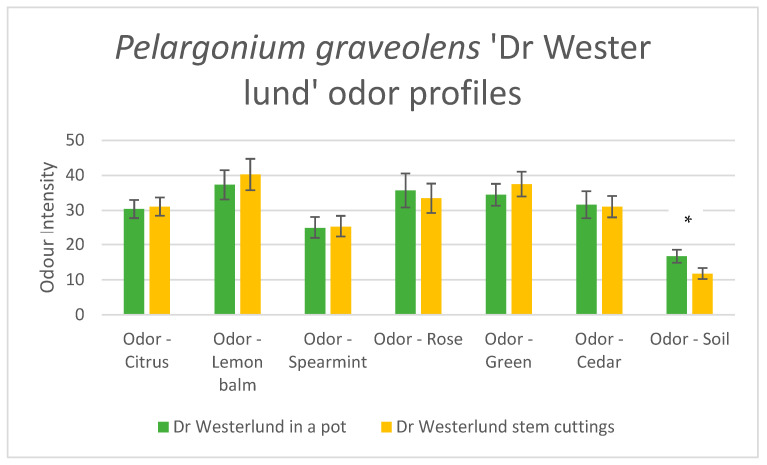
Sensory odor profiles of *Pelargonium graveolens* ‘Dr. Westerlund’ measured on the sensory intensity scale. Green reflects the whole potted plant and yellow represents the stem cuttings. The * indicates the statistically significant difference between *Pelargonium graveloens* ‘Dr Westerlund’ in a pot and *Pelargonium graveloens* ‘Dr. Westerlund’ stem cuttings in the attribute soil.

Figure 1 shows the sensory odor profiles of the samples as mean values from the panelists’ assessments; error bars show the standard error of means. The profiles show distinct odor characteristics of citrus, lemon balm, spearmint, rose, green odors, and cedar. The one and only characteristic differing between the samples was soil, marked in the figure by *. This difference was expected since the soil appeared only in the potted plant sample; the difference was statistically significant, *p* < 0.05.

### 2.2. Chemical Analysis

The chemical compounds of the *Pelargonium graveolens* ‘Dr. Westerlund’ found in the headspace and identified by the GC-MS and the MassHunter Analysis 8.0 Software ibrary are presented in Table 1. Moreover, the retention times, retention indexes, and the Chemical Abstracts Service (CAS) numbers are provided. 

### 2.3. Sensory and Chemical Analysis

The sensory attributes, along with the sensory definitions and suggested chemical explanations, are provided in Table 2. Both sensory and chemical analyses show that the only difference between samples is the soil odor, which appears for samples “in a pot” and not for stem cuttings and leaves. Furthermore, the odor descriptors of the proposed chemical compounds are shown in the table, including these descriptors’ origin from the MS library and from the literature. For identification, the CAS numbers are also given. Most of the identified chemical compounds are commonly used in different food applications.

The resulting data show that mainly citrus, lemon, and campherous volatile compounds were found to be attributed to the specific odor of *Pelargonium graveolens* ‘Dr. Westerlund’. Further, Alpha-Guaiene was identified as being attributed to the earthy odor.

## 3. Discussion

This study aimed to develop the sensory profile describing the perception and to find suggestions for the correlating chemical compounds of rose-scented *Pelargonium graveolens* ‘Dr. Westerlund’ as a potted plant and stem cuttings by sensory and chemical analyses. The intention is to use these results as an input for a future study, where the chemical compounds will be quantified and to a higher extent be correlated to the sensory results as well as to health effects. It has been shown by Pálsdóttir et al. [17] that the *Pelaronium graveolens* ‘Dr. Westerlund’ elicited positive emotions and memories, as well as reduced the perceived stress among patients suffering from stress-related mental disorders. The results are supported by earlier findings, e.g., Shirzadegan et al. [24], who illustrated decreased anxiety when using the geranium essential oil, and Matsumoto et al. [25], who demonstrated that the distinct notes of citrus and floral scent could support stress reduction. Findings such as these highlight the need to increase the understanding of the underlying factors explaining these perceived benefits and the physiological reaction that might reduce stress. The impact of the volatiles on stress is to be further investigated when quantifying the chemical compounds found in *Pelargonium graveolens* ‘Dr. Westerlund’. This study contributes by analyzing the sensory experiences of *Pelargonium graveolens* ‘Dr. Westerlund’ by using a trained sensory panel, resulting in a sensory profile. Such a profile, to our knowledge, has not been published elsewhere. The presented sensory profile may function as an important tool, not only in a health-related context, but it may also be highly important for horticultural stakeholders in developing consumer-oriented descriptive terms when communicating plants’ unique odor compositions and how these are linked to health and well-being. This may also be of importance for the odor/scent industry in the development of new products, such as essential oils and hydrolates, for the support of human health and well-being.

In connection with the evaluation conducted by the analytical sensory panel, the volatile compounds in *Pelargonium graveolens* ‘Dr. Westerlund’ were then identified by GC-MS analyses to determine a possible molecular background of the perceived odors and thereby form a foundation for future investigations concerning whether these compounds could be responsible for the perceived well-being. The volatile compounds identified in this study are in line with previous studies, such as those by Taherpour et al. [26], Rajeswara Rao et al. [27], and Dabiri et al. [28] who analyzed volatile oil extracts from different varieties of pelargoniums. The most common use of these extracts is essential oils, but only a few studies have used natural fragrances from plants as the current one, e.g., Sowndhararajan and Kim [29]. In the present study, quantification was not possible in the chosen experimental set-up, and it should be noted that variations in the chemical composition may occur due to seasonal changes, geographical location, growing and weather conditions, etc. [30,31,32].

Table 2 suggests the chemical compounds that are attributed to and elicit the perceived sensory experience of *Pelargonium graveolens* ‘Dr. Westerlund’. As previously described, plant odors are often complex, comprising a great variety of aromatic compounds [33,34], which, as a consequence, contributes to the difficulty in securely linking chemical compounds to specific odors. Therefore, the presented results and suggestions are to be considered an important contribution to the human experience of natural odor, specifically those elicited by *Pelargonium graveolens* ‘Dr. Westerlund’. The suggestions were the results of comparisons of, on the one hand, the sensory definitions and, on the other hand, descriptions of volatiles in the ‘Handbook of Flavor Ingredients’ [22] and the MS library. Surprisingly, and although the specific mass numbers for geraniol were used, the substance could not be identified in the sample; this would have been expected, as other studies have shown this to be a common compound in the odor of many pelargonium species. However, there are seasonal variations in the volatile compounds in all types of pelargonium species, as well as large differences in the amount of geraniol in flowers and other parts of the plant, where the flowers contain the largest amount. The highest yield of volatile compounds is normally found during the summer period [35]. In this study, stem and leaves were used, and the plants were cultivated and sold in a greenhouse environment. These factors may be explanations for the nondetectable amount of geraniol. Further, it may be noted that in an acidic environment, geraniol may convert to the cyclic terpene α-terpineol [36]. 

By using internal standards, both the quantitative and qualitative identification would improve; however, the analysis in this study was conducted to find possible chemical candidates for an explanation of the sensory profile. For a full explanation, a gas chromatography–olfactometry (GC-O) could be another successful way to analyze the volatiles [37]. GC-O is a powerful method for the analysis of sensorially perceivable volatile components, by the inclusion of both a chemical analysis in the form of gas chromatography and sensory assessors who analyze each component that is seen in a chromatogram for its odor characteristics [38]. Only a few studies, if any, have analyzed both the chemical constituents of fresh, or oil from, pelargonium varieties and the perceivable odor of the volatiles. Nonetheless, there are some studies that list the aroma characteristics, but these studies have not performed any sensory analyses themselves; instead, they use the literature for this purpose, e.g., Gauvun et al. [39].

As mentioned, the studies by Pálsdóttir et al. [17], Fekri et al. [40], and Shirzadegan et al. [24] reported decreased stress and anxiety in humans as a result of pelargonium volatiles. Lis-Balchin and Roth [41] showed that geranium oil could be applied for muscle relaxation. Other studies have shown the origin of bioactive compounds from a diverse group of pelargoniums that may be used in different areas [41,42]. For example, monoterpenes are potentially effective for pest management [42]; methyl eugenol is microbiologically active and may be used in food preservation and in household products [41]. Phytol, sabinene, β-caryophyllene, linalool, and camphor are known for their analgesic and anti-inflammatory potentials [30]. Essential oils from rose-scented geranium have a wide range of usages, e.g., they are commonly used in the cosmetic and perfumery industry [40], but they can also be effectively used to reduce anxiety [24,43]. The use of a living plant material is not as common but could be an alternative to achieve stress reduction, as it also gives the benefits of handling and taking care of the plant [43]. This could be especially important in nature-based therapies for humans [44,45,46,47] or for the public in their everyday life [10]. A recent study reveals that the perception of odor pleasantness is universal, as it can be predicted by the physiochemical properties of the molecules, rather than the cultural aspects [48]. This means that the findings of the current study could apply to a larger population than just the Swedish population recovering from stress-related mental disorders.

## 4. Materials and Methods

### 4.1. Samples

For the sensory and chemical analysis, altogether 16 samples of the plant *Pelargonium graveolens* ‘Dr. Westerlund’ were used for the analysis. The plants were propagated and sold by a local grower in the south Sweden region, produced in early winter and harvested during the fall. All plants were only exposed to growing conditions in greenhouses and were not exposed to outdoor settings with natural daylight and varied temperatures. 

The samples were put into plastic buckets with lids. A resealable hole in the lid was opened by the assessor to take a deep sniff for the sensory evaluation. The samples were from *Pelargonium graveolens* ‘Dr. Westerlund’:

1. Plants in a pot with soil
2. Stem cuttings with leaves

The samples were put in buckets for a minimum of two hours before the analysis to concentrate the volatile compounds in the headspace.

### 4.2. Sensory Analysis

The group making up the sensory panel was selected and trained according to the ISO (International Standard Organization) standard 8586-2:2008. The panel comprised nine assessors who were trained to perform a quantitative descriptive analysis, which is an analytical sensory method for developing sensory profiles of products in a non-subjective way. During training, reference samples were presented, and the sensory descriptors were developed and defined in consensus by the panelists (see Table 1). During the first out of two training sessions, the first step was to present two samples of *Pelargonium graveloens* ‘Dr Westerlund’, one in a pot and one with stem cuttings and leaves. The samples were kept in buckets with lids in which a small part could be opened to take a sniff. The nine assessors took their own notes on their individual perceptions of the odors. As a second step, the panelists gathered and decided in consensus which descriptors should be used; a definition of each descriptor was then developed. Reference examples, such as different citrus fruits and a piece of cedar wood, were available to help the assessors to reach consensus in the attribute definitions.

During the second training session, the panel trained on how to rate descriptor intensities, see ISO 13299:2016 (Sensory analysis–Methodology–General guidance for establishing a sensory profile). In this study, an unstructured line scale was anchored with weak at 5% of the scale and strong at 95% of the scale [49]. At the end of the training session, all 16 *Pelargonium graveloens* ‘Dr Westerlund’ plants were included and presented in plastic buckets as described above, either in a pot or as stem cuttings and leaves (equaling one plant). Thereafter, the assessments were performed in duplicate, in randomized order. The panelists were instructed to sniff and breathe in the arm fold to neutralize the senses. The sensory assessments were performed in a sensory laboratory equipped according to the ISO standard, see ISO 8589-2007 (general guidance for the design of test rooms). The software Eye Question (Version 5.0.7.11, Elst, The Netherlands) was used for sensory data collection.

Each assessor signed up for participation after being informed about the products and the terms of participation: voluntary participation, freedom to leave the test without giving a reason, and the right to refrain from answering specific questions. The procedure followed the Swedish Ethics Review Act, which applies to research carried out in Sweden and if the research includes processing of sensitive personal data. This study includes questions about odor perception, which, according to the Data Protection Ordinance, are not classified as sensitive personal data. According to the General Data Protection Regulation (GDPR), no responses to any of the questionnaires used in this study include information that can be traced to or used to identify any individual.

### 4.3. Chemical Analysis

Headspace sampling of the biogenic volatile organic compounds (BVOC) was performed using solid phase microextraction (SPME). Sampling at ambient temperature (20 °C) was carried out in two steps: First, headspace sampling from leaves (weighing about 2 mg) that were rolled and placed in 15 mL vials, and second, from plants placed in 30 l plastic buckets, where three replicates were performed. To find the most suitable sampling time, 5, 10, and 40 min were tested. Based on assessing the peak height of the chromatograms, the sampling time for the vials was 10 min, and for the buckets, 40 min. To control the emission of volatiles from the plastic material in the buckets, blank sampling was performed. The SPME holder, the fibers–50/30 μm (DVB/CAR/PMDS), and the 15 mL vials were all purchased from Supelco (Bellafonte, PA, USA). The fibers were conditioned according to the supplier’s instructions prior to their use. The SPME fiber with the sample was inserted directly into the GC injector for thermal desorption for 10 min and was then removed. The GC/MS instrument was an Agilent 7890B, coupled with an Agilent 5977B mass spectrometer (MS) (Agilent Technologies, Inc., Santa Clara, CA, USA). The GC was equipped with an HP-5ms ultra inert column (length 30 m). Flow was set to 1.5 mL/min with helium gas, and the inlet was programmed to 230 °C, splitless mode. The oven was programmed as follows: initial 40 °C hold for 4 min, temperature ramp, rate 6 °C/min, end at 230 °C, and hold for 2 min. Mass spectra were generated in the electron ionization (EI) mode at 70 eV, with the scan range of *m*/*z* 40–300 and a scan rate of 100 spectra/s. The temperature of the quadrupole was 150 °C, and the ion source temperature was 230 °C. Data were processed using Agilent MassHunter Analysis 8.0 (Agilent, Santa Clara, CA, USA).

### 4.4. Data Evaluation

The scales used in the sensory analysis were converted into numerical numbers corresponding to a scale running from 0 to 100. Sensory data were analyzed by calculating descriptive statistics such as the mean values, standard deviations, standard errors, etc. Means and standard deviations are shown in Section 2. The data were further subjected to a two-way analysis of variance (ANOVA), with samples and measurements as fixed effects. Significant differences (*p* < 0.05) were calculated between the samples via the Tukey’s post hoc pairwise comparison test (IBM SPSS, version 26).

Chemical data were subjected to the Mass Hunter Analysis 8.0 Software library; where possible, chemical compounds were suggested. Further, the suggested compounds were compared to the literature sources [22] and databases [23] to ensure identification. This was conducted qualitatively by picking those compounds described similarly in the literature and by the panelists. The sensory results and descriptions were then compared with the resulting chemical compounds, and suggestions were made for the chemical compounds responsible for the sensory experience. Retention times and retention indexes are given for the suggested compounds.

## 5. Conclusions

This study provides new knowledge and suggested explanations for the odor of *Pelargonium graveloens* ‘Dr Westerlund’. The main findings and results of the presented study provide sensory profiles of *Pelagonium graveolens* ‘Dr. Westerlund’ as a potted plant and as stem cuttings, as well as suggestions for chemical compounds that are attributed to the sensory profiles. The following suggestions were reported: limonene (citrus odor), para-cymene (lemon balm), camphene (spearmint), 4-Methyl-2-(2-methylprop-1-en-1-yl), tetrahydro-2H-pyran (rose), citronellyl formate (green), camphene (cedar), and alpha-guaiene (soil). For the sensory profiles, the one and only difference between potted plants and the stem cuttings was the odor of soil. Further studies are recommended to investigate the odor experience of the scented pelargonium species in relation to stress reduction in humans, measuring the neural activity of the brain when exposed to chemical compounds of *Pelargonium graveolens* ‘Dr. Westerlund’. Further, we suggest a study where the odor experience is simultaneously investigated in combination with gas chromatography, e.g., gas chromatography–olfactometry. It is of importance to understand the relation between odors and human health and well-being, and this study identifies the sensory profile of *Pelagonium graveolens* ‘Dr. Westerlund’, that scent has been recognized as stress reducing.

## Figures and Tables

**Table 1:** Chemical compounds found in the *Pelargonium graveolens* ‘Dr. Westerlund’ samples.

Name: Chemical Compound	Retention Time (Minutes)	Retention Index	Formula: Chemical Compound	CAS Number
alpha-pinene	14.30	1447	C_10_H_16_	80-56-8
Camphene	14.81	1476	C_10_H_16_	79-92-5
Para-cymene	16.87	1580	C_10_H_14_	99-87-6
Alpha -terpineol	16.98	1585	C_10_H_18_O	98-55-5
Limonene	16.99	1793	C_9_H_13_NO_3_	5989-27-5
Cyclohexanol	17.92	1678	C_12_H_22_O	108-93-0
4-Methyl-2-(2-methylprop-1-en-1-yl)tetrahydro-2H-pyran	19.12	1696	C_10_H_18_O	3033-23-6
Cyclohexanone	20.31	1757	C_10_H_18_O	108-93-0
Citronellol	21.71	1829	C_10_H_20_O	106-22-9
Citronellyl formate	22.80	1882	C_11_H_20_O_2_	105-85-1
Copaene	25.49	2366	C_15_H_24_	3856-25-5
Caryophyllene	26.50	2445	C_15_H_24_	13877-93-5
Alpha-Guaiene	26.75	2464	C_15_H_24_	3691-12-01
(−)-Aristolene	26.93	2478	C_15_H_24_	6831-16-9
Aromadendrene	27.17	2496	C_15_H_24_	72747-25-2
Humulene	27.21	2499	C_15_H_24_	6753-98-6
3 furopelargone A	29.67	2690	C_15_H_22_O_2_	1143-45-9

**Table 2:** Sensory attributes, their definitions, and suggested chemical compounds. Chemical formulas and CAS numbers are given in Table 1.

Sensory Attribute	Definition ^1^	Chemical Compound ^2^	Description ^3,4,5^
Citrus	Citrus fruit, including peel	Limonene	Citrus, orange, fresh, sweet ^3,5^ Lemon-like odor, free from campherous and terpene notes ^4^
Lemon balm	The whole plant	Para-cymene	Fresh, Citrus, Terpene, Woody, Spicy ^3,5^ Citrusy aroma and reminiscent of lemon ^4^
Spearmint	Odor of green mint	Camphene	Camphoraceous, cooling, piney, woody with terpy nuances. It has citrus and green minty and green spicy notes ^3,5^ Terpene, camphorous flavor ^4^
Rose	Rose water	4-Methyl-2-(2-methylprop-1-en-1-yl)tetrahydro-2H-pyran	Fragrance found in roses and rose oil ^3^ Rose oxide, powerful geranium top note ^4^
Green	Meadow with grass and nettles	Citronellyl formate	Sweet, green, waxy, floral, apricot, citrus, fruity and mandarin ^3^ Fruity and rose-like odor ^4^
Cedar	Cedar tree	Camphene	Camphoraceous, cooling, piney, woody with terpy nuances ^3^ Terpene, camphorous flavor ^4^
Soil	Soil	Alpha-Guaiene	Sweet, woody, dry guaiac wood, spicy, powdery ^3,5^ Earthy and spicy aroma and woody flavor ^4^

^1^ Sensory Definition used by the analytical panel; ^2^ Identified by GC-MS; ^3^ MS-library; ^4^ Handbook of Flavor Ingredients [22]; ^5^ The Good Scents Company [23].

## Data Availability

Data are available upon request.
